# Deep cervical lymphatic-venous anastomosis in dementia: a clinical and mechanistic evaluation

**DOI:** 10.1097/JS9.0000000000004561

**Published:** 2025-12-17

**Authors:** Cong Tang, Xiaoju Zheng, Haijun Li, Baoshan Wang, Yuqi Zheng, Wenbin Song, Aimei Wu, Linjun Xin, Dengwen Zhang, Rongguo Yang, Shuang Du, Peng He, Yujiao Li, Linjuan Wu, Xinrong Wang, Qi Shi, Gonçalo J.L. Bernardes

**Affiliations:** a Xi’an Fengcheng Hospital, Xi’an, Shaanxi, China; b GIMM - Gulbenkian Institute for Molecular Medicine, Lisboa, Portugal; c Nova School of Business and Economics, Carcavelos, Portugal; d Yusuf Hamied Department of Chemistry, University of Cambridge, Cambridge, UK; e Translational Chemical Biology Group, Spanish National Cancer Research Centre 26 (CNIO), Madrid, Spain

**Keywords:** Alzheimer’s disease, central nervous system clearance, cognitive recovery, deep cervical lymphatic-venous anastomosis, dementia, glymphatic system, immune modulation, meningeal lymphatics, neurodegeneration, Tau protein

## Abstract

**Background:**

Alzheimer’s disease and other dementias are marked by progressive cognitive decline, with few effective treatments available in advanced stages. Impaired clearance of neurotoxic proteins such as amyloid-beta and Tau is a central pathological feature. Deep cervical lymphatic-venous anastomosis (dcLVA) is a novel microsurgical approach designed to enhance cerebrospinal fluid (CSF) clearance via extracranial lymphatic pathways, potentially enabling removal of neurotoxic proteins.

**Objective:**

To evaluate the clinical effects and potential mechanisms of dcLVA in patients with advanced dementia, focusing on cognitive, functional, and immunopathological outcomes.

**Methods:**

Twenty-eight patients with advanced dementia underwent dcLVA. Cognitive performance was assessed using the Mini-Mental State Examination (MMSE), alongside evaluations of bowel and bladder control, emotional stability, behavioral responsiveness, and self-care abilities. Biological parameters – including CSF protein, tumor markers, lymphocyte counts, and peripheral blood concentrations of Aβ1-42 and phosphorylated Tau-181 (P-tau181) – were measured pre- and postoperatively. Immunohistochemical analysis of resected cervical lymph nodes was performed to detect CNS-derived Tau protein.

**Results:**

Within 1 week after dcLVA, 64.3% of patients demonstrated MMSE improvement, with further functional gains observed in continence, emotional regulation, motor coordination, and feeding independence. Patients with circulatory comorbidities were more likely to show cognitive recovery. Tau protein was detected in all examined cervical lymph nodes, providing direct histopathological evidence of CNS-derived pathological protein transport via the lymphatic system. In a subset of cognitively improved patients, peripheral lymphocyte counts normalized. In contrast, tumor markers and CSF total protein showed no significant correlation with outcomes.

**Conclusions:**

dcLVA may promote cognitive and functional recovery in patients with dementia by enhancing CNS waste clearance and modulating systemic immune responses. These exploratory findings describe postoperative changes observed in this cohort; however, the single-arm design precludes causal interpretation.

## Introduction

Dementia is a heterogeneous clinical syndrome characterized by progressive cognitive decline, neurobehavioral impairment, and loss of functional independence. Affecting over 50 million people globally – a number projected to double by 2050 – it presents a major public health challenge^[1]^. Alzheimer’s disease (AD) is the most common form, accounting for 60–70% of cases^[2]^. Despite varying etiologies among subtypes such as vascular, frontotemporal, and Lewy body dementia, a shared hallmark is the accumulation of neurotoxic proteins such as β-amyloid and hyperphosphorylated Tau, leading to synaptic failure, neuroinflammation, and widespread neurodegeneration^[3]^.

Current therapeutic approaches such as cholinesterase inhibitors and NMDA receptor antagonists offer only transient symptomatic benefits and do not address underlying disease mechanisms^[4,5]^. In advanced stages, treatment becomes predominantly supportive, with significant burden on caregivers and healthcare systems. Recently, monoclonal antibodies targeting amyloid-beta (Aβ), such as lecanemab and donanemab, have shown promise in slowing disease progression by promoting the clearance of pathological aggregates^[6]^. However, their clinical use is limited by modest efficacy, high cost, the risk of amyloid-related imaging abnormalities, and challenges in delivering therapeutics across the blood–brain barrier^[7]^. As such, there is an urgent need for disease-modifying strategies that can enhance pathological protein clearance, regulate neuroimmune interactions, and improve neurological function.

Emerging evidence has identified the meningeal lymphatic system and the glymphatic pathway as critical components of brain homeostasis^[8–11]^. These systems facilitate the clearance of interstitial solutes, including Aβ and Tau proteins, from the central nervous system (CNS) into peripheral lymphatic vessels. Experimental studies in animal models have demonstrated that dysfunction in these pathways contributes to the accumulation of neurotoxic metabolites and is associated with cognitive decline^[10,12–14]^. Recent reviews also highlight circadian and hormonal regulation of glymphatic dynamics, including the impact of sleep–wake cycles and menstrual physiology, on CNS clearance capacity^[15–17]^. Moreover, perioperative factors such as anesthesia and surgical stress have been shown to influence glymphatic function, underscoring the relevance of clearance pathways in surgical contexts^[18]^. Conversely, enhancing meningeal lymphatic outflow has been shown to reduce protein burden, modulate neuroinflammation, and improve neurological outcomes in models of AD and traumatic brain injury^[10,14]^.

The deep cervical lymph nodes are a key drainage site for CNS-derived solutes via the meningeal lymphatic system. However, whether surgical augmentation of cervical lymphatic outflow can influence neurological recovery in human dementia remains largely unexplored. Deep cervical lymphatic-venous anastomosis (dcLVA) is a microsurgical technique originally developed to relieve peripheral lymphedema by establishing direct outflow between lymphatic vessels and adjacent veins. Its potential role in modulating intracranial clearance pathways and neurological outcomes has only recently begun to be investigated.

In this study, we evaluated the clinical effects of dcLVA in 28 patients with dementia who underwent dcLVA, with a focus on cognitive, autonomic, emotional, and functional outcomes. We further explored potential mechanisms by evaluating associations between dcLVA and changes in systemic biomarkers, peripheral lymphocyte profiles, and CNS-derived pathological proteins. By integrating clinical observations with immunohistochemical (IHC) and biochemical analyses, we aim to investigate whether surgical enhancement of extracranial lymphatic drainage could offer therapeutic benefit for patients with dementia, particularly those with limited treatment options in advanced disease stages.

This cohort study has been reported in line with the STROCSS guidelines^[19]^.

## Methods

### Study design and patient selection

This prospective study was initiated at Xi’an Fengcheng Hospital, China in May 2024 to explore the efficacy and safety of dcLVA for the treatment of severe dementia. Patients were eligible if they had a confirmed diagnosis of dementia and an MMSE score ≤ 20. Disease severity at baseline was further classified using the Clinical Dementia Rating (CDR). By December 2024, a total of 28 patients underwent the surgical intervention, the majority of whom were classified as CDR = 3 (severe dementia), with a smaller number at mild (n = 2) or moderate (n = 1) stages.

**HIGHLIGHTS**

- First clinical report of deep cervical lymphatic-venous anastomosis (dcLVA) performed in 28 patients with advanced dementia, demonstrating early and measurable improvements in cognition, motor coordination, continence, and self-care.
- Systematic evaluation of factors associated with cognitive recovery post-dcLVA, identifying circulatory comorbidities and lymphocyte normalization as potential predictors of benefit.
- First immunohistochemical demonstration of CNS-derived Tau protein in cervical lymph nodes from human patients, providing direct histopathological evidence of extracranial clearance of neurotoxic proteins.

### Ethics statement

This study was conducted in accordance with the Declaration of Helsinki and approved by the Ethics Committee of Xi’an Fengcheng Hospital (Approval number: JS2024-001-01). All participants had advanced dementia with very limited therapeutic options. Surgical eligibility was confirmed by multidisciplinary evaluation involving neurology and cardiology teams to ensure safety and exclude major contraindications.

Because some patients at this stage were unable to provide meaningful informed consent, written informed consent was obtained from their legal guardians or next-of-kin. Families were fully informed of the experimental nature of the procedure, the potential risks, and the absence of established efficacy. The decision to proceed was made jointly with family members under strict ethical oversight, taking into account the lack of alternative therapies, the severity of disease, and the substantial caregiver burden.

All dcLVA procedures were performed before the July 2025 prohibition issued by the National Health Commission. No operations occurred after the ban, and the study was designed for scientific exploration rather than clinical treatment.

### Patient inclusion and exclusion criteria

Patients were enrolled based on the following inclusion criteria:

1) Age between 50 and 90 years, with a confirmed diagnosis of dementia made by a tertiary hospital;

2) Evaluation by both neurology and cardiology departments confirming the absence of severe cardiovascular or coagulation disorders;

3) Mini-Mental State Examination (MMSE) score ≤ 20;

4) Strong willingness for intervention expressed by family members, with inclusion of patients across mild, moderate, and severe stages based on clinical judgment.

Exclusion criteria were as follows:

1) Unclear diagnosis or comorbid neurological disorders that may affect cognitive function;

2) Presence of severe cardiovascular or coagulation disorders precluding safe surgical intervention;

3) Family members with unrealistic expectations or excessive demands regarding surgical outcomes.

### Patient demographics and clinical characteristics

Among the 28 patients enrolled, 11 were male, and 17 were female. Patients predominantly exhibited severe neuropsychiatric symptoms, urinary and fecal incontinence, and sleep disturbances. Dementia subtypes included AD diagnosed in 23 patients (82%), vascular dementia in two patients, frontotemporal dementia in two patients, and dementia with Lewy bodies in one patient. Baseline demographic and clinical data, including age, disease duration, psychiatric symptoms, and comorbidities, were recorded systematically prior to surgical intervention (Supplemental Digital Content Figure 1, available at: http://links.lww.com/JS9/G468 **and** Supplemental Digital Content Patient Information, available at: http://links.lww.com/JS9/G489).

**Figure 1.**
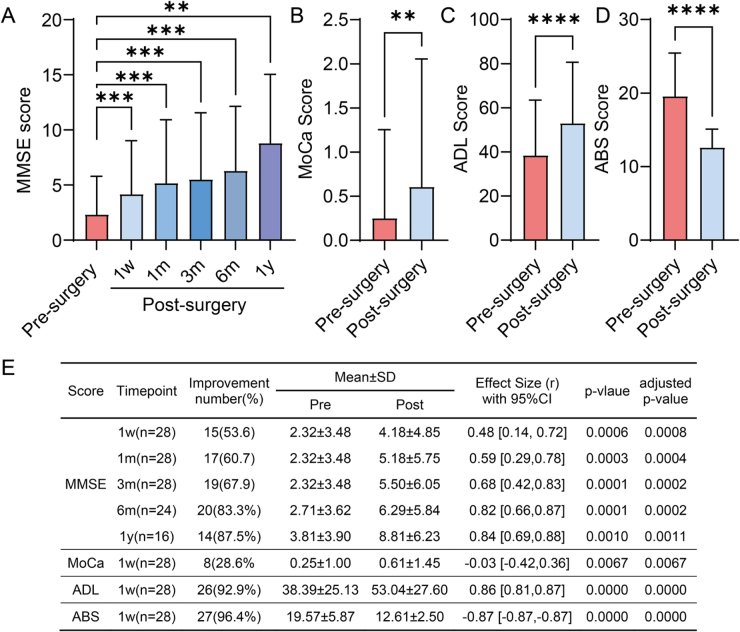
Postoperative cognitive changes following deep cervical lymphatic-venous anastomosis. A) Individual MMSE scores before surgery and at postoperative follow-ups in patients with dementia: pre-surgery (n = 28), 1 week (n = 28), 1 month (n = 28), 3 months (n = 28), 6 months (n = 24), and 12 months (n = 16). No imputation (e.g., LOCF) was applied; analyses were conducted on available cases. A progressive increase in MMSE scores was observed in patients who completed serial assessments, indicating a trend of sustained cognitive improvement. B) MoCA scores at pre-surgery and 1 week postoperatively, showing significant improvement. C) ADL scores at pre-surgery and 1 week postoperatively, showing significant improvement. D) ABS scores at pre-surgery and 1 week postoperatively, showing a significant reduction. E) Quantitative summary of MMSE, MoCA, ADL, and ABS results (mean ± SD), including r effect sizes, p-values and FDR-adjusted *P*-values. Statistically significant improvements were detected as early as 1 week after surgery, with further gains at 1 and 3 months on the MMSE (*P* < 0.01, Wilcoxon signed-rank test with FDR correction). Data at 6 and 12 months showed continued upward trends, though based on smaller sample sizes. Missing data at later time points were due to loss to follow-up or inability of some patients to complete cognitive assessments because of fluctuating clinical status. ABS, Agitated Behavior Scale; ADL, Activities of Daily Living; MMSE, Mini-Mental State Examination; MoCA, Montreal Cognitive Assessment.

### Surgical procedure

All surgical procedures were performed under general anesthesia with ultrasound guidance. Indocyanine green (ICG, 0.1 ml) was injected subcutaneously around the jugular foramen region to visualize lymphatic structures. A longitudinal incision was made through the skin and subcutaneous tissue along the posterior border of the platysma muscle, extending from just below the mandibular angle to approximately 1.0 cm inferior to the cricoid cartilage.

The platysma muscle was carefully exposed and retracted anteriorly, revealing the posterior platysmal fascia. Upon opening this fascia, adipose tissue and cervical lymph nodes became visible. Using ICG fluorescence imaging, lymph nodes were assessed: enlarged or malformed nodes lacking fluorescence were excised, while healthy-appearing lymph nodes demonstrating strong fluorescence, elasticity, and clearly visualized fluorescent lymphatic vessels were preserved for subsequent anastomosis.

Selected lymph nodes were anastomosed to suitable small branches of the external jugular vein or directly to the external jugular vein itself in an end-to-side fashion. Additionally, small cervical veins exhibiting minimal backpressure were anastomosed to fluorescent lymphatic vessels by either end-to-end or sleeve-type techniques. Following meticulous hemostasis and wound irrigation, a negative-pressure drainage tube was placed. The incision was then closed in layers with sutures.

A supplementary video demonstrating the key steps of the dcLVA procedure has been provided (Supplemental Digital Content Video 1, available at: http://links.lww.com/JS9/G467).

### Postoperative care and follow-up

All patients received standardized postoperative care, including pain management, wound care, and regular assessment for surgical complications. Postoperative evaluations of cognitive, emotional, and functional status were performed systematically at multiple time points (1 week, 1 month, 3 months, and 6 months). Follow-up assessments included cognitive testing with the MMSE, MoCA (cognitive domains), ADL (functional capacity), and ABS (behavioral symptoms), clinical evaluation of bowel and bladder control, emotional and sleep assessments, and peripheral blood biomarkers measurements. All cognitive and functional assessments were performed by trained and certified neuropsychological evaluators; however, blinding to time points was not implemented.

### Pathological and biomarker analyses

Cervical lymph nodes excised during surgery were analyzed using immunohistochemistry. Peripheral blood samples were collected preoperatively and postoperatively to assess AD-related biomarkers. Additionally, lymphocyte counts and cerebrospinal fluid (CSF) protein levels were systematically recorded and analyzed to explore potential relationships with cognitive outcomes. CSF samples from 23 patients were also collected and sent to the laboratory for testing of AD-related biomarkers, including Aβ1-40, Aβ1-42, the Aβ1-42/Aβ1-40 ratio, P-Tau-181, and total Tau.

### IHC analysis

IHC staining was performed on formalin-fixed, paraffin-embedded cervical lymph node sections obtained during dcLVA surgery. Tissue sections were deparaffinized, rehydrated, and subjected to antigen retrieval using 20 × citrate buffer (pH 6.0, Servicebio, G1202) at high temperature. Endogenous peroxidase activity was quenched using 3% hydrogen peroxide for 25 minutes at room temperature, followed by blocking with 3% BSA for 30 minutes. Sections were incubated overnight at 4°C with a rabbit polyclonal anti-Tau antibody (Anti-Tau Rabbit pAb, Servicebio, GB111608-50) at the manufacturer’s recommended dilution. After washing with PBS, slides were incubated for 50 minutes at room temperature with HRP-conjugated goat anti-rabbit IgG secondary antibody (Servicebio, GB23303, 1:200). DAB chromogen (Servicebio, G1212) was used for color development, and hematoxylin was applied for nuclear counterstaining. Slides were dehydrated through graded ethanol, cleared in xylene, and mounted with neutral resin. Positive staining appeared brown in the cytoplasm and/or perinuclear regions, while nuclei were counterstained blue. Images were examined and captured under a Nikon E100 bright-field microscope.

### Statistical analysis

Statistical analyses were performed using GraphPad Prism (version 9.0, GraphPad Software, San Diego, CA, USA) and Python (version 3.11) with the SciPy library (version 1.15.0; scipy.stats). Continuous variables are presented as mean ± standard deviation (SD). Normality was tested using the Shapiro–Wilk test. Because the data do not follow a normal distribution, pairwise comparisons with the preoperative baseline were performed using the Wilcoxon signed-rank test with False Discovery Rate (FDR) correction. Effect sizes were reported using Rosenthal’s r derived from the standardized Wilcoxon signed-rank test statistics. Categorical variables are reported as frequencies and percentages. Associations between two dichotomous categorical variables (e.g., cognitive improvement and potential influencing factors) were evaluated using the phi coefficient (φ) with 95% confidence intervals derived via Fisher’s z transformation. A two-sided *P*-value of < 0.05 was considered statistically significant.

## Results

### Cognitive function improvement

One week after the procedure, all 28 patients completed the assessment. Cognitive performance was assessed using the MMSE, Montreal Cognitive Assessment (MoCA), Activities of Daily Living (ADL) and Agitated Behavior Scale (ABS) preoperatively and at multiple postoperative time points (Fig. 1A**–**D). Normality testing with the Shapiro–Wilk test showed non-normal distributions at pre-surgery and 1 week; therefore, Wilcoxon signed-rank test were used with FDR correction. Statistical analysis showed that all postoperative scores were higher than baseline, compared to preoperative levels, with statistically significant differences (Fig. 1E). Among them, 15 patients (53.6%) showed postoperative increases in MMSE scores, with a mean MMSE increase of 3.5 points (range: 1–9), while 13 patients (46.4%) exhibited no significant change from baseline. Results from other functional assessments also indicated positive changes: in the MoCA score, eight patients (28.6%) showed postoperative increases, with a mean increase of 0.36 points; in the ADL score, 26 patients (92.9%) showed score increases with a mean change of 14.64 points; and in the ABS score, 27 patients (96.4%) showed that the score decreases with a mean change of 6.96 points. MMSE demonstrated moderate to large effect sizes at all time points, indicating sustained improvement in cognitive function. ADL and ABS both showed large effect sizes, reflecting substantial functional gains and marked reductions in behavioral symptoms. In contrast, MoCA consistently exhibited a small effect size, suggesting that the impact of surgery on this measure was relatively limited.

At the 1- and 3-month follow-ups, MMSE were available for all 28 patients, and at the 6-month and 1-year follow-up, data were available for 24 and 16 patients. Missing follow-up assessments were mainly due to loss to follow-up or the patient’s inability to complete cognitive testing because of severe fluctuations in clinical condition. The mean MMSE increases at each time point were 2.86 points, 3.18 points, 3.58 points, and 5.00 points, indicating a trend of progressive cognitive enhancement over time.

### Other clinical improvements

#### Bowel and bladder control

Among the 28 patients, four (#2, #10, #11 and #28) had normal bowel and bladder function preoperatively and remained normal postoperatively. Postoperative recovery of bowel and bladder function was evaluated in 24 patients with complete preoperative and postoperative data (Supplemental Digital Content Table 1, available at: http://links.lww.com/JS9/G468). Sustained continence changes were noted at 6 months in two patients (#3 and #5), while additional four patients (#7, #8, #12, and #13) maintained recovery through at least 3 months. An additional 14 patients demonstrated postoperative continence increases at the one-month follow-up but did not sustain gains at subsequent time points, suggesting transient or fluctuating recovery trajectories. In total, 20 of 24 patients (83.3%) experienced continence recovery at or beyond the 1-month mark.

Patients #3 and #5, both of whom presented with preoperative disorientation, exhibited sustained improvements in autonomic function and consciousness, suggesting broader neurological recovery. Patient #13, who was preoperatively unconscious, regained function with a delayed but sustained response through three months. Notably, patients #22 and #23, both of whom were preoperatively unconscious with preserved walking ability, showed early postoperative continence changes and may represent cases of motor and autonomic system co-recovery.

Rapid functional responses were also recorded: patient #21 voluntarily requested toilet use just 18 minutes after surgery, and 15 of the 24 patients (62.5%) achieved continence within 24 hours postoperatively. This is an isolated observation that may reflect postoperative fluctuation or caregiver-related factors rather than a solely direct surgical effect. Conversely, two patients (#14 and #20) showed no continence improvement during the documented follow-up period.

These findings, while striking, indicate that dcLVA may contribute to early and, in selected cases, sustained recovery of bowel and bladder function in patients with severe preoperative neurological impairment. However, the high proportion of transient recoveries underscores the need for extended follow-up to assess the durability of these effects, as well as require confirmation in larger and controlled studies. These rapid postoperative observations are presented solely as anecdotal reports and should not be interpreted as consistent or reproducible effects.

#### Emotional status

All 28 patients (100%) showed postoperative changes in emotional status within 3 days post-surgery, including enhanced alertness and responsiveness. Emotional fluctuations were observed in four male patients during the recovery period; notably, one patient experienced two distinct episodes of emotional instability within 2 months postoperatively. These fluctuations may be associated with individual psychological conditions or environmental factors, highlighting the importance of ongoing psychological monitoring and support to ensure sustained emotional stability post-surgery.

#### Sleep quality

Postoperative changes in sleep quality were noted in 14 of 15 patients who were evaluated, with patients showing increased total sleep time, fewer nighttime awakenings, and improved daytime alertness. Nevertheless, sleep disturbances re-emerged in three patients between one to 5 months postoperatively, suggesting variability in long-term sleep recovery. Additionally, two patients who were dependent on sedative medications before surgery continued the same dosage regimen postoperatively and exhibited hypersomnia symptoms; therefore, improvements in sleep and cognition in these cases may have been confounded by pharmacological effects rather than surgery alone. These findings highlight the potential for persistent pharmacological dependence and the importance of individualized medication management during postoperative care.

#### Additional functional changes

Other notable postoperative changes included complete resolution of hoarseness and choking symptoms in all four patients who had reported these issues preoperatively. Two patients who required passive feeding preoperatively transitioned successfully to active feeding, indicating recovery of autonomous feeding abilities. Additionally, eight patients exhibited significant improvements in limb stiffness and gait abnormalities, with movements becoming more natural and flexible. Collectively, these enhancements significantly contributed to improved overall quality of life.

### Influence of clinical and biological factors on postoperative cognitive recovery

To explore potential factors associated with cognitive improvement after dcLVA, patients were stratified according to preoperative comorbid conditions and biomarker profiles.

#### Comorbid conditions

**Figure 2.**
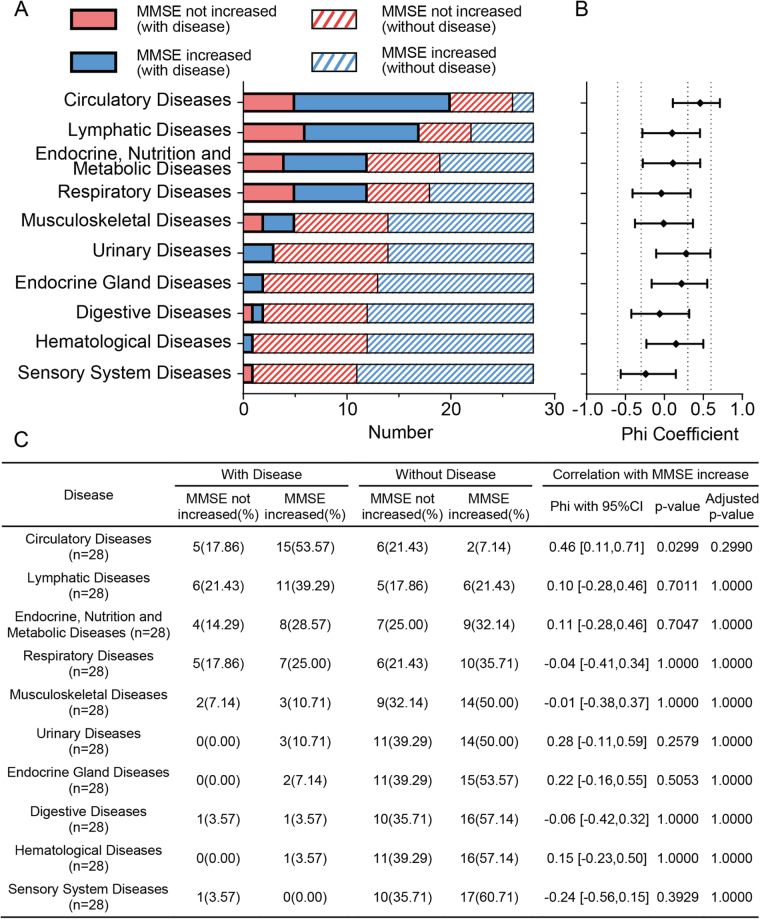
Association between systemic comorbidities and postoperative cognitive improvement in 28 patients. A) Distribution of patients by comorbidity type and cognitive outcome (MMSE increased vs Not increased). Colored bars distinguish disease presence and MMSE outcomes. B) Phi coefficients with 95% confidence interval representing the strength of association between each disease category and postoperative MMSE improvement. C) The summary table shows the number and percentage of patients with or without preexisting diseases, further stratified by MMSE outcome. MMSE, Mini-Mental State Examination.

To explore the potential impact of systemic comorbidities on postoperative cognitive recovery, we analyzed MMSE score 1 month changes in 28 patients stratified by preoperative comorbid disease categories (Fig. 2A). Among all systemic conditions evaluated, circulatory diseases were the most prevalent, observed in 71.4% (20/28) of patients. Patients with circulatory diseases demonstrated markedly higher rates of cognitive improvement following intervention, when compared to those without such comorbidities. Specifically, among patients with circulatory diseases, 15 out of 20 (75%) exhibited increased MMSE scores, whereas only two out of eight (25%) of those without circulatory diseases showed such improvement. This indicates that patients with circulatory comorbidities were nearly three times more likely to demonstrate cognitive improvement than those without. A moderate positive association (|φ| = 0.46) was also observed between circulatory comorbidities and postoperative cognitive improvement. However, after adjustment, the *P*-value did not reach statistical significance (adjusted *P* = 0.2990), indicating a potential false positive. Nevertheless, there remains a potential link between circulatory comorbidities and postoperative cognitive improvement that warrants further investigation (Fig. 2B**–**C). No statistically significant associations were found for other systemic disease categories, including lymphatic, endocrine-metabolic, respiratory, musculoskeletal, urinary, or digestive systems (all *P* > 0.05) and their lower phi coefficients (|φ| < 0.3) indicate minimal or negligible associations with cognitive improvement.

#### Tumor marker status

**Figure 3.**
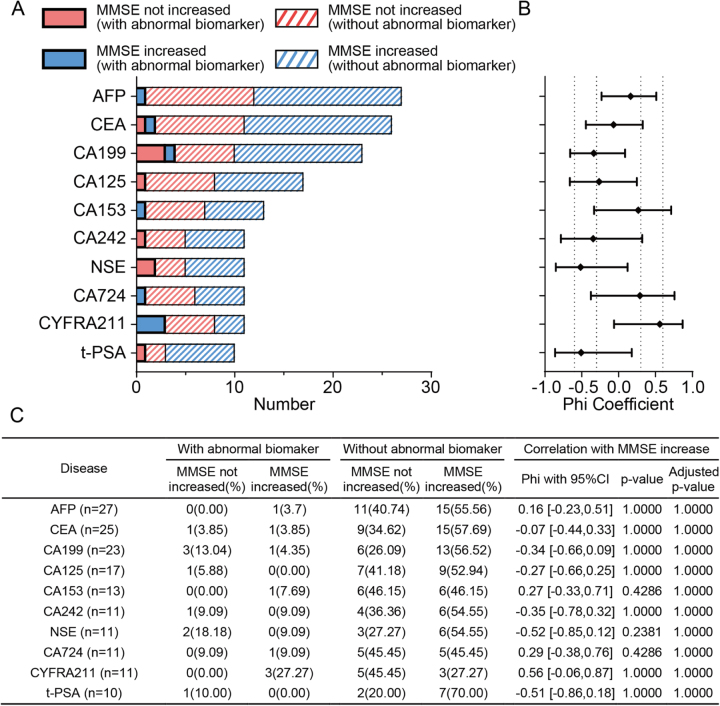
Association between tumor biomarker abnormalities and postoperative MMSE improvement. A) Distribution of patients by tumor biomarker status and cognitive outcome (MMSE increased vs Not increased). Colored bars distinguish biomarker abnormality and MMSE outcomes. A total of 10 tumor markers were assessed, including AFP, CEA, CA199, CA125, CA153, CYFRA21-1, NSE, CA724, CA242, and t-PSA. B) Phi coefficients with 95% confidence interval representing the strength of association between each tumor biomarker abnormality and postoperative MMSE improvement. None of the biomarkers showed a statistically significant correlation (all *P* > 0.05), indicating weak or no association between tumor marker abnormalities and cognitive outcome. C) The summary table shows the number and percentage of patients with or without abnormal biomarkers, further stratified by MMSE outcome. AFP, alpha-fetoprotein; CEA, carcinoembryonic antigen; MMSE, Mini-Mental State Examination; NSE, neuron-specific enolase; t-PSA, total prostate-specific antigen.

To assess the potential relationship between tumor marker levels and postoperative cognitive recovery, we analyzed changes in MMSE scores stratified by preoperative tumor marker status (Fig. 3). No statistically significant association was identified between the presence of tumor marker abnormalities and MMSE improvement. For all tumor markers evaluated, *P*-values exceeded 0.05. Although some tumor markers showed phi coefficients greater than 0.3, none of the unadjusted *P*-values reached statistical significance (all *P* > 0.05), indicating a negligible correlation between tumor marker positivity and postoperative cognitive outcomes.

#### CSF tests

**Figure 4.**
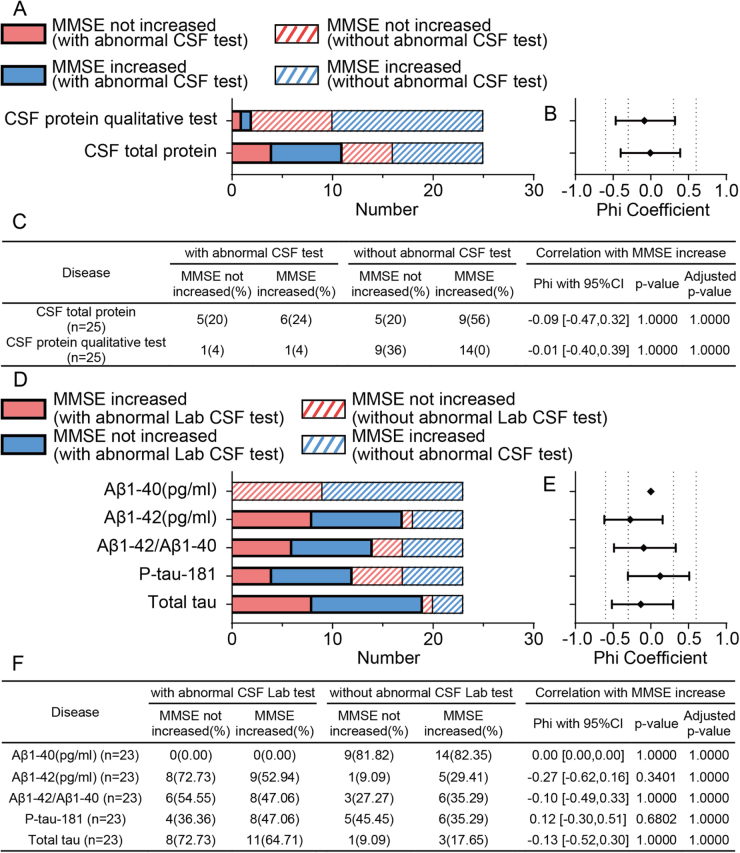
Association between CSF tests and postoperative MMSE improvement. A) Distribution of patients by CSF tests and cognitive outcome (MMSE increased vs Not increased). Colored bars distinguish CSF protein abnormalities and MMSE outcomes. Patients were categorized based on CSF total protein concentration and CSF protein qualitative test results. B) Phi coefficients with 95% confidence interval representing the strength of association between each CSF test and postoperative MMSE improvement. None of the CSF tests showed a statistically significant correlation (all adjusted *P* = 1.0000), indicating weak or no association between these CSF test and cognitive improvement. C) The summary table shows the number and percentage of patients with or without abnormal CSF tests, further stratified by MMSE outcome. D) Distribution of patients according to CSF Alzheimer’s disease (AD)-related biomarkers (Aβ1-40, Aβ1-42, Aβ1-42/Aβ1-40 ratio, P-tau181, total Tau) and MMSE outcome. E) Phi coefficients with 95% confidence intervals showing the association between each biomarker and MMSE improvement. F) Summary table of patients stratified by abnormal biomarker status and MMSE outcome. None of the CSF biomarkers demonstrated statistically significant associations with cognitive recovery (all adjusted *P* = 1.0000), indicating minimal or negligible prognostic value. Aβ, amyloid-beta; AD, Alzheimer’s disease; CSF, cerebrospinal fluid; MMSE, Mini-Mental State Examination; P-Tau, phosphorylated Tau.

We further examined whether CSF parameters were associated with postoperative cognitive recovery. Neither CSF total protein concentration nor qualitative protein status showed a significant correlation with MMSE improvement. Patients with abnormal and normal CSF total protein levels exhibited nearly identical rates of cognitive improvement. The corresponding phi coefficient was approximately 0.04, with all *P*-values equal to 1.0000, indicating no predictive value of CSF total protein for postoperative cognitive improvement. (Fig. 4A**–**C).

CSF samples from 23 patients were also sent to the laboratory for testing of AD relevant indicators. Similarly, none of the CSF biomarkers, including Aβ1-40, Aβ1-42, the Aβ1-42/Aβ1-40 ratio, P-Tau-181, or total Tau, showed statistically significant associations with cognitive recovery. All biomarkers had very low phi coefficients (|φ| < 0.3), indicating minimal or negligible associations. These findings indicate that the evaluated CSF biomarkers provide little to no prognostic insight into postoperative cognitive recovery (Fig. 4D**–**F).

#### Lymphocyte count dynamics

**Figure 5.**
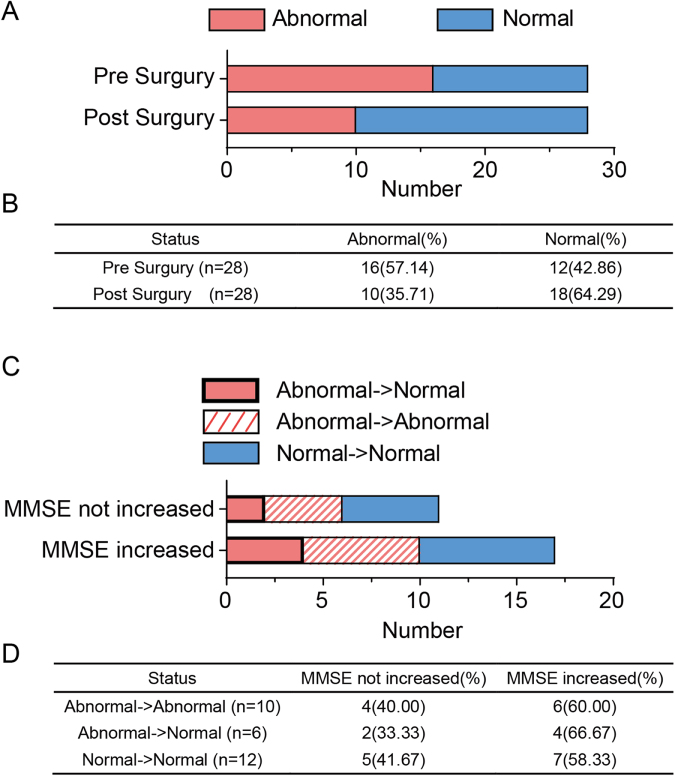
Association between normalized peripheral blood lymphocyte count and postoperative improvement in MMSE scores. A) Bar plots showing peripheral blood lymphocyte status (normal vs abnormal) before and after surgery. A shift toward normal lymphocyte levels was observed postoperatively. B) The summary table shows the distribution and percentage of patients with normal or abnormal lymphocyte status before and after surgery. C) Bar plots stratifying patients by changes in lymphocyte status: from abnormal to normal, persistently abnormal, and consistently normal, and their corresponding MMSE improvement status following dcLVA. D) The summary table shows the number and percentage of patients with or without MMSE improvement in each lymphocyte transition group. dcLVA, deep cervical lymphatic-venous anastomosis; MMSE, Mini-Mental State Examination.

Preoperative lymphocyte abnormalities were observed in 57.1% (16/28) of patients, while 42.9% (12/28) had normal lymphocyte counts. Following dcLVA, the proportion of patients with abnormal counts decreased to 35.7% (10/28), and the number of patients with normal counts increased to 64.3% (18/28), suggesting a trend toward immune profile normalization (Fig. 5). However, chi-square analysis showed that this trend was not statistically significant (*P* = 0.1803).

To assess whether lymphocyte normalization was associated with cognitive improvement, subgroup analysis was performed based on MMSE outcomes. Among patients whose MMSE scores did not improve after surgery (n = 10), only two patient’s lymphocyte count recovered from abnormal to normal. In contrast, among patients whose MMSE scores improved (n = 18), four patients whose preoperative abnormal lymphocyte counts recovered to normal levels (abnormal to normal). Still, chi-square testing did not reveal a statistically significant association (*P* = 0.9419).

These comparisons suggest that postoperative normalization of lymphocyte profiles may be associated with cognitive improvement, particularly in patients with preoperative immune abnormalities. Although statistical significance was not formally tested due to the small data size, the observed trend supports further exploration of immune modulation as a potential mechanism underlying cognitive recovery following dcLVA.

### Detection of pathological proteins in cervical lymph nodes

**Figure 6.**
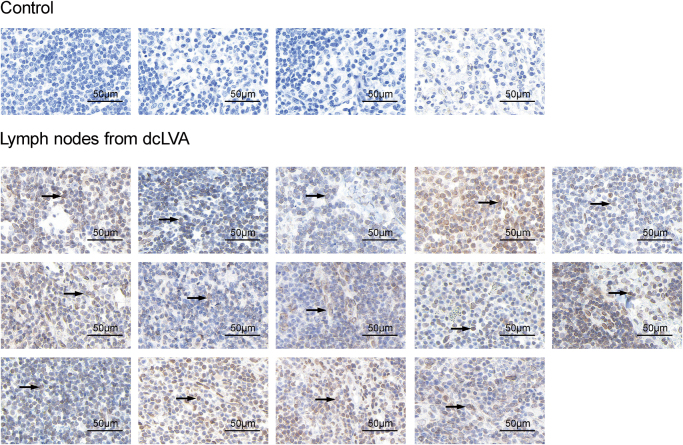
Immunohistochemical detection of Tau protein in cervical lymph nodes resected during dcLVA. IHC images demonstrating Tau protein expression in lymph node tissues obtained from patients who underwent dcLVA. Control tissues were axillary lymph nodes obtained from breast cancer patients undergoing routine surgical excision and showed minimal or no detectable Tau staining. In contrast, all lymph nodes resected during dcLVA exhibited variable levels of Tau protein positivity. Black arrowheads indicate representative areas of Tau-positive staining. All scale bars represent 50 μm. dcLVA, deep cervical lymphatic-venous anastomosis; IHC, immunohistochemistry.

To investigate the potential involvement of cervical lymphatic pathways in the clearance of CNS-derived pathological proteins, immunohistochemical (IHC) staining was performed on lymph node specimens obtained during dcLVA surgery in 14 patients. Phosphorylated Tau protein, a hallmark of AD pathology, was detected in all examined samples (Fig. 6). These findings demonstrate the presence of AD-associated protein aggregates within the deep cervical lymphatic system. The consistent detection of CNS-derived Tau in peripheral lymphatic tissues provides pathological evidence supporting a possible anatomical route through which dcLVA may contribute to the clearance of neurotoxic proteins in patients with dementia.

### Changes in peripheral AD biomarkers following dcLVA

Peripheral blood concentrations of Aβ1–42 and phosphorylated Tau-181 (P-tau181), biomarkers commonly associated with AD, were measured in a subset of eight patients with dementia both preoperatively and on postoperative day 5 to assess short-term biochemical changes following dcLVA. A general decline in Aβ1–42 levels was observed in most patients. For example, concentrations decreased from 116.48 to 92.95 pg/mL in one case, and from 148.84 to 69.89 pg/mL in another, indicating potential postoperative shifts in amyloid metabolism (Supplemental Digital Content Table 2, available at: http://links.lww.com/JS9/G468).

In contrast, P-tau181 levels exhibited heterogeneous responses. Some patients showed postoperative increases (e.g., 15.42 to 32.69 pg/mL), while others demonstrated notable declines (e.g., 47.72 to 20.30 pg/mL), suggesting individual variability in Tau-related dynamics.

Although preliminary, these findings imply that dcLVA may influence peripheral biomarker profiles in patients with dementia. However, the small sample size, with blood Aβ and P-tau181 available in only eight patients, and limited follow-up period preclude definitive interpretation. Further longitudinal studies are required to validate these trends and assess their clinical significance.

### Safety

No major complications or procedure-related mortality occurred. Minor postoperative issues were observed, including transient wound swelling (common, with 7–8 cases noted as more pronounced), wound pain in three patients (resolved within 2 months), and delayed wound healing in two patients (#18 and #12), both of which ultimately healed after local care. One patient (#15) experienced postoperative emotional fluctuations, which improved with supportive management. According to the Clavien–Dindo classification, all observed adverse events were grade I–II (Supplemental Digital Content Table 3, available at: http://links.lww.com/JS9/G468).

## Discussion

This exploratory study describes multidimensional postoperative functional changes in patients with dementia after dcLVA, including improvements in cognition, continence, emotion, sleep quality, and mobility. Among the most notable findings, more than half of patients demonstrated cognitive improvement as early as 1 week after surgery, with sustained gains observed in a subset up to 6 months. These results support the hypothesis that enhancing extracranial lymphatic drainage may modulate the neurobiological environment of the brain and facilitate recovery, even in late-stage disease^[8,9]^. Given the single-arm before-after design, these observations represent associations rather than definitive treatment effects.

Enrollment of severely demented individuals was justified under a last-resort, exploratory framework given the absence of effective alternatives. Legally authorized guardians provided consent under full disclosure, and oversight was ensured by the institutional ethics committee.

Postoperative changes in bowel and bladder control were also observed in over 80% of evaluated patients, with a subset showing sustained recovery and concurrent gains in consciousness and mobility. The restoration-like postoperative changes of autonomic function, in some cases within 24 hours, support the hypothesis that dcLVA may alleviate neurogenic dysfunction through enhanced clearance of brain-derived metabolic waste or modulation of neuroinflammation^[20]^. Postoperative stabilization of emotional status and resolution of neurovegetative symptoms further point toward a systemic regulatory effect, likely through a combination of neuroimmune and perfusion-related pathways.

Subgroup analyses identified circulatory comorbidities as a potential clinical predictor of better cognitive outcomes. Patients with preoperative circulatory comorbidities exhibited disproportionately higher rates of cognitive improvement. These findings may reflect a compensatory effect, whereby impaired vascular-mediated solute clearance in patients with circulatory comorbidities enhances their responsiveness to interventions that promote alternative pathways, such as lymphatic drainage. Prior studies have indicated that reduced arterial pulsatility impairs glymphatic flow, and surgical enhancement of lymphatic outflow may partially compensate in such cases^[21]^. These patients may also harbor more reversible metabolic or perfusion-related cognitive deficits, rather than irreversible neurodegeneration, further contributing to their observed postoperative gains^[22,23]^. Additionally chronic low-grade inflammation common in cardiovascular disease may also be alleviated by improved lymphatic drainage^[10]^. In contrast, tumor marker status and CSF total protein levels were not predictive of cognitive outcomes, reinforcing the limited relevance of these non-specific biomarkers in this context^[24]^.

The detection of phosphorylated Tau in all resected cervical lymph nodes provides histopathological evidence that CNS-derived proteins can transit into the peripheral lymphatic system. This finding aligns with preclinical models suggesting that interstitial solutes, including β-amyloid and Tau, drain via glymphatic and meningeal lymphatic pathways into cervical lymphatics^[8,10]^. These observations parallel findings from animal models demonstrating impaired glymphatic and meningeal lymphatic clearance in aging and neurodegeneration, and are consistent with human MRI studies showing reduced perivascular clearance and lymphatic dysfunction in dementia^[9,10,12,13,15–17]^. Whether this reflects passive drainage or active immune-mediated transport remains unclear, but the presence of Tau protein in peripheral nodes directly supports the mechanistic rationale for dcLVA. However, this observation alone does not establish causality, and further studies will be required to determine whether dcLVA directly enhances clearance or clinical benefit. Complementing this, peripheral blood levels of Aβ1-42 showed consistent postoperative reductions in some patients, while P-tau181 responses varied across individuals. These biomarker changes further support the hypothesis that dcLVA may facilitate the removal of pathological proteins involved in neurodegeneration. This finding is suggestive but not conclusive and does not prove direct clearance from the brain, as alternative sources cannot be excluded.

Notably, postoperative normalization of peripheral lymphocyte profiles was more frequently observed among cognitively improved patients, raising the possibility that immune modulation may play a mechanistic role in the therapeutic effects of dcLVA^[25]^. Although the limited sample size precludes statistical confirmation, this trend supports the concept of brain-immune system crosstalk and its relevance in dementia pathophysiology. Impaired meningeal lymphatic flow has been linked to systemic immune dysregulation and neuroinflammation^[26]^, and restoration of this flow could help reestablish immune homeostasis.

From a behavioral and psychosocial perspective, the recovery process was shaped not only by physiological changes but also by environmental context. Several patients showed fluctuations in MMSE scores and atypical behaviors, such as mirror misrecognition or toileting disorientation, underscoring the importance of consistent environments, caregiver training, and structured rehabilitation. Encouragingly, spontaneous behaviors like singing and improved hygiene autonomy were observed, suggesting recovery of higher-order circuit function including frontotemporal and limbic integration. Nonetheless, some of the observed functional improvements (e.g., feeding, toileting) may also have been influenced by attentive caregiving, and thus should be interpreted with caution as exploratory observations rather than definitive surgical effects.

Collectively, these findings highlight the therapeutic potential of targeting the cervical lymphatic system in the management of dementia. While causality cannot be established in this preliminary study, the observed functional, immunological, and pathological changes suggest a novel intervention pathway that warrants further mechanistic exploration. These findings are also aligned with emerging perspectives integrating computational intelligence and predictive modeling into clinical neuroscience and surgical innovation, as highlighted by recent analyses^[27]^. Despite these promising findings, this study has limitations.

### Limitations

This exploratory single-arm study lacks a control group, includes a small sample size with attrition, and used non-blinded assessments, all of which limit interpretation and may introduce bias. Observed changes should therefore be interpreted cautiously.

The sample size was small, and no prospective sample size calculation was performed because the study of dcLVA was exploratory and data to estimate effect sizes and statistical power were limited. A post hoc bootstrap analysis (5000 resamples) using paired Wilcoxon signed-rank tests was performed to estimate statistical power. The results of MMSE improvement indicated a large effect size (*r* = 0.88, rank-biserial correlation) with >99% power at α = 0.05, suggesting the primary finding is unlikely due to chance. However, the small sample, incomplete biomarker data, and short follow-up restrict the generalizability of the results. Future randomized trials with predefined sample sizes and longer follow-up are needed.

Functional and cognitive outcomes were assessed without blinding, which may have introduced observer bias; future studies should incorporate blinded evaluations to strengthen validity. Follow-up durations were limited, and the absence of a control group precludes definitive attribution of observed effects to dcLVA alone. In addition, attrition was substantial and many improvements were transient rather than sustained, which may lead to overrepresentation of positive responders. As a result, natural disease fluctuation, placebo effects, and the influence of attentive caregiving cannot be excluded and may have contributed to the observed changes. Our study did not include direct neuroimaging or CSF flow assessments (e.g., MRI, ASL, PET), which limits mechanistic interpretation. These modalities were not feasible in this exploratory pilot study due to cost constraints and the frailty of patients with advanced dementia, but they will be important in future investigations. Future randomized trials with standardized outcome metrics, longitudinal neuroimaging, and multi-omic biomarker profiling are necessary to further elucidate mechanisms and identify optimal candidates for surgical intervention.

In summary, dcLVA appears to offer a novel therapeutic approach for patients with dementia by promoting clearance of pathological proteins, modulating immune responses, and improving functional connectivity. This lymphatic-based intervention may fill a crucial gap in the treatment of patients with advanced disease stages, where pharmacologic options are limited. However, given the single-arm design, small sample size, limited follow-up, and absence of a control or matched comparison, these findings should be interpreted as exploratory and hypothesis-generating rather than definitive evidence of efficacy. Future controlled studies with larger cohorts, longer follow-up, and integration of neuroimaging and biomarker analyses will be necessary to validate these preliminary observations and to clarify the true therapeutic potential of targeting the cervical lymphatic system in dementia.

## Data Availability

The datasets generated and analyzed during the current study are not publicly available due to patient privacy and ethical restrictions but are available from the corresponding author on reasonable request.

## Research Registration Unique Identifying Number (UIN)

researchregistry11346 https://researchregistry.knack.com/researchregistry#home/registrationdetails/686ab1ad10eccb02fef6fb51/.

## Data Sharing Statement

Anonymized data are available upon reasonable request and require approval from the institutional ethics committee. Only de-identified datasets can be shared, and requests should be directed to the corresponding author.
